# Chemical Gastroenterocolitis after Dental Root Canal Therapy with Camphorated and Mentholated Chlorophenol

**DOI:** 10.1155/2021/9918830

**Published:** 2021-06-28

**Authors:** Mikheil Kalandarishvili, Ernst-Wolfgang Kolbe, Günther Winde, Michael Kaspari

**Affiliations:** University Clinic of General, Abdominal and Thoracic Surgery, Hospital of Herford, Herford, Germany

## Abstract

A 78-year-old man with a history of pancolitis, after the treatment of dental abscess with oral antibiotics and local application of camphorated and mentholated chlorophenol (CMCP), presented with abdominal pain of 4-day duration, as well as hair loss in the area of moustache and finger nail lifting. He was already treated with rectal application of budesonide because of pancolitis, diagnosed 6 weeks ago and interpreted as an allergic reaction to clindamycin. For further investigation, we performed gastroscopy and colonoscopy, which showed the edematous mucosa with polypus-like changes of the whole mucosa of the stomach, duodenum, first part of the jejunum, distal ileum, complete colon, and rectum. The diagnosis was complicated and was achieved in synopsis with anamnestic details, such as endodontic application of camphorated chlorophenol. The patient symptoms abated after he commenced on mesalazine therapy.

## 1. Case Presentation

A 78-year-old man was admitted in our surgical unit on January 2020 with lower abdominal pain of 4-day duration as well as constipation. He complained about unintended weight loss, loss of performance, loss of hair in the area of moustache, and also detachment of 3 fingernails during the last 2 months. Medical history from a third party was unremarkable. Hematologic and biochemical blood tests including serum electrolytes, liver enzymes, amylase, and renal function parameters showed no abnormalities. The C-reactive protein level was normal at 3.6 mg/L (normal range (NR): < 5 mg/L), but white blood cell count was increased to 29.7 × 10^9^/L (NR: 4. 5–11 109/L) due to elevation of neutrophil count at 24.5 × 10^9^/L (NR: 2–7 × 10^9^/L) with slight elevation of the eosinophil level at 1.63 × 10^9^/L (NR: <0.4 × 10^9^/L), and the protein level was slightly decreased at 55.6 G/L (NR: 66.0–87.0 G/L). Stool cultures for bacteria and viruses showed negative findings.

An abdominal CT scan was significant for previously known small left adrenal mass (remained unchanged compared to the previous examination) and small reducible incisional hernia in the area of midline laparotomy after right hemicolectomy, which was performed 5 years ago because of colon cancer. There was no sign of any changes in the gastrointestinal (GI) tract. The colon cancer was diagnosed in February 2015 with stage I, classified using the American Joint Committee on Cancer, so that there was no need for adjuvant cancer therapy. Until now, the regular cancer follow-up was unremarkable.

**Figure 1:**
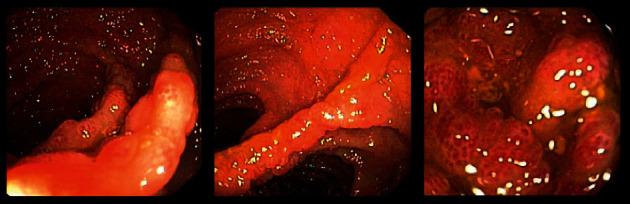
Colonoscopy 14 days after treatment of tooth abscess.

The patient referred himself 6 weeks ago in gastroenterology (GE) of our hospital with diarrhea of 12-day duration, which was manifested 2 days after surgical treatment of tooth abscess. At that time, his dentist incised the abscess in the area of central incisors 8 and 9, inserted camphorated and mentholated chlorophenol (CMCP) in the root canal, and prescribed the oral antibiotic clindamycin. After diarrhea manifestation in 2 days, clindamycin was rejected, and CMCP was once again locally reinserted. Diarrhea progressed despite cancelling of antibiotics, and his performance status worsened further. However, the initial colonoscopy performed in the GE unit showed the polypoid-hemorrhagic pancolitis (Figure 1). Biopsies taken from the terminal ileum, sigmoid, and rectal mucosa showed the distorted crypt architecture as well as eosinophilic infiltration, so the allergic reaction was suggested. Primary short-term therapy with intravenous cortisone relieved his symptoms. After that, rectal application of budesonide for 8 weeks was prescribed, and the patient was discharged.

**Figure 2:**
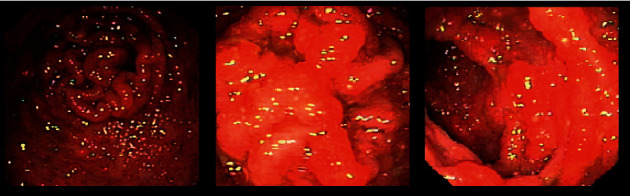
Colonoscopy 8 weeks after treatment of tooth abscess.

**Figure 3:**
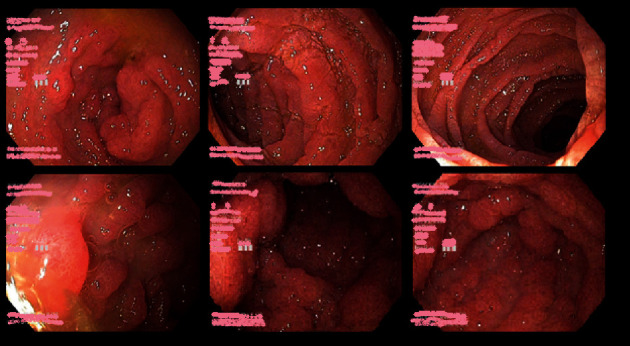
Gastroscopy 8 weeks after treatment of tooth abscess.

For further investigation, we performed esophagogastroduodenoscopy and colonoscopy with multiple biopsies. Endoscopic investigation showed that the whole mucosa of the stomach, duodenum, first part of the jejunum, distal ileum, complete colon, and rectum were edematous with polypus-like changes of the all aforementioned parts of the GI tract (Figures 2 and 3). Histological studies showed the ubiquitous unspecific inflammation, atrophy of the villi, and regenerative changes of all the samples collected in all the parts of the GI tract. There were no eosinophilic collections any more.

Subsequently, he was started on mesalazine to facilitate symptom relief and intestinal healing. There was an improvement in his clinical symptoms with respect to abdominal pain, and inflammation markers were declining.

Follow-up colonoscopy two months later showed no significant improvement, but there were no further changes seen, which were not grossly observed on prior colonoscopy. Over the next few months, the patient had gradual deterioration of his performance status and reported a further weight loss, hair loss, and nail loss. After this, the patient obtained parenteral nutrition. The GE of our hospital implemented the individual monitoring strategy for the patient with clinical, blood marker, and endoscopy assessment with intervals of 3 months. His further treatment was performed in gastroenterology of another university hospital at his own request.

## 2. Discussion

There are many types of inflammations of separate parts of the digestive tract. The followed forms of gastritis are described in literature: autoimmune, bacterial, chemical, Crohn-associated, eosinophilic, lymphocytic, radiation, and noninfectious granulomatous gastritis [1]. The common types of colitis are as follows: inflammatory bowel disease (ulcerative colitis, Crohn's disease, and indeterminate colitis) [2], microscopic (lymphocytic and collagenous colitis) [3], radiation-induced, infectious (pseudomembranous colitis caused by Clostridium difficile, enterohemorrhagic colitis caused by Shiga toxin, etc.) [4, 5], chemotherapy-induced [6], diversion [7], ischemic, checkpoint inhibitor induced, [8] and chemical colitis [9]. Even though most of the aforementioned medical conditions are single-organ diseases and have preferential areas of location, it can be, in some cases, expected that other parts of the gastrointestinal (GI) system or distant organs are also affected, for example, duodenal manifestation of Crohn's disease [10], pseudomembranous gastritis [11], and backwash ileitis in ulcerative colitis [12]. Gastroenteritis or enteritis is a general name of the condition, which is mostly infectious and characterized with diarrhea or vomiting [13].

The GI inflammation manifested in all parts of the GI system is rarely described; also, the definition “gastroenterocolitis” (GEC) is rarely used in the literature. There is no uniform classification or definition of this condition. After comprehensive search in the literature, we have identified the following types of GEC: fulminating *Staphylococci* GEC [14], PD-1 inhibitor GEC [8], nosocomial rotavirus-induced GEC [15], collagenous GEC [16], and diffuse infectious phlegmonous GEC [17].

Onycholysis is a common medical problem, which is associated with the painless separation of the nail from the nail bed. It can be a sign of many different conditions, including trauma, manicuring, psoriasis, nail fungus, phototoxic dermatitis due to drugs, hyperthyroidism, sarcoidosis, amyloidosis, connective tissue disorders, vitamin or iron deficiency, and chemotherapy [18]. The literature review showed us that clindamycin has an extremely low potential to induce a drug-induced photosensitivity [19].

Hair loss, also known as alopecia, is a very common condition. There are many types of alopecia, which is subdivided into two main categories.

### 2.1. Nonscarring Alopecia

- Androgenetic alopecia
- Alopecia areata
- Telogen effluvium
- Traction alopecia
- Trichotillomania
- Anagen effluvium

### 2.2. Scarring Alopecia

- Alopecia mucinosa
- Metastatic infiltrate
- Favus

The pathophysiology of hair loss is predominantly well understood and depends on the alopecia type. In androgenetic alopecia, both factors, hormones and genetics, play a role in the pathogenesis. Alopecia areata is not well understood, but the most common hypothesis involves autoimmunity as a T-cell-mediated pathway. In telogen effluvium, stress and hormones are mainly described triggers. Traction alopecia is primarily caused by pulling force being applied to the hair. Trichotillomania is a mental disorder characterized with pulling out of hair. Chemotherapeutic aggression is the cause of anagen effluvium, and the infiltration of the scalp with abnormal lymphocytes causes alopecia mucinosa. Tinea capitis (favus) is dermatophytic infection responsible for alopecia [20]. There is a subtype of alopecia areata of the beard known as alopecia barbae. It is limited exclusively to the beard and characterized with losing beard hair in small circular patches [21].

Considering the etiopathogenesis of all the aforementioned diseases, the inflammation of the GI system in our case is most likely the chemical origin, and the hair and nail loss can also be explained with the chemical impact of toxins. We believe that the aforementioned changes are due to the immunotoxic effect of CMCP. We also considered the side effect or allergy to clindamycin, but it appears less feasible as the patient had a past history of 1-week antibiotics with clindamycin approximately 10 years ago. On the contrary, the selective hair loss in the area of the upper lip indicates the local effect of toxins (in our case, CMCP). Moreover, in the poison control center in Bonn, Germany, one alopecia case has already been reported after treatment with CMCP. The toxicity of camphorated chlorophenol in the animal model is also established [22].

Also, there are many similarities with the rare disease Cronkhite-Canada syndrome (CCS), which is manifested with ubiquitous gastrointestinal polyps, associated with diarrhea and malabsorption [23]. The malabsorption further leads to alopecia and onycholysis. As the first symptom of our patient was hair loss in the area of the upper lip, it is very unlikely that this was a random manifestation of a very rare disease CCS. The stool culture must be done regularly in order to exclude an antibiotic-induced colitis or viral infection.

CMCP is an antibacterial agent that is used in the treatment of dental root canal systems. The common reported side effect of chlorophenol is its toxicity, but the side effects of camphorated chlorophenol are extremely rare [24]. To the best of our knowledge and literature review, this is the first reported case of GI tract inflammation associated with the use of this medication. The use of CMCP is a widely spread dental treatment in Germany with few adverse effects reported. Because of the lack of data, it is not feasible to assess the possible risk factors for the development of the described symptom complex. Other alternative antimicrobial medicines for root canal treatment are, for example, chlorhexidine gel 2%, chlorhexidine powder 1%, povidone-iodine, and polyhexanide [24].

## 3. Conclusion

We experienced a case in which an oral application of CMCP caused a massive chemical inflammation of the GI tract. The chemical ignition of the GI system is rare and usually limited to one organ. The diagnosis can be challenging if the pertinent history is not obtained, and this case illustrates a delay in diagnosis. Therefore, patients with unexplained inflammation of the GI tract must be questioned about the past dental treatment. We should also bear in mind that the ingestion of CMCP can cause alopecia and nail lifting. The literature is not clear about the therapy of chemical GEC, and in our case, mesalazine was, in an initial phase, a useful treatment, but subsequently, it was not successful and encouraging. The purpose of this case report is to raise awareness about this uncommon adverse effect of CMCP.
